# 76 Methods and Perceptions of Success for Patient Recruitment in Decentralized Clinical Trials – ADDENDUM

**DOI:** 10.1017/cts.2023.593

**Published:** 2023-07-28

**Authors:** Brian Miyata, Barbara Tafuto, Nadina Jose

The above abstract [1] published without listing Brian Miyata as an author.

The original article has been corrected online to rectify this omission.
